# No Effect of Genome-Wide Copy Number Variation on Measures of Intelligence in a New Zealand Birth Cohort

**DOI:** 10.1371/journal.pone.0055208

**Published:** 2013-01-30

**Authors:** Andrew T. M. Bagshaw, L. John Horwood, Youfang Liu, David M. Fergusson, Patrick F. Sullivan, Martin A. Kennedy

**Affiliations:** 1 Department of Pathology, University of Otago, Christchurch, New Zealand; 2 Department of Psychological Medicine, University of Otago, Christchurch, New Zealand; 3 Department of Genetics, University of North Carolina at Chapel Hill, North Carolina, United States of America; The Roslin Institute, University of Edinburgh, United Kingdom

## Abstract

Variation in human intelligence is approximately 50% heritable, but understanding of the genes involved is limited. Several forms of genetic variation remain under-studied in relation to intelligence, one of which is copy number variation (CNV). Using single-nucleotide polymorphism (SNP) -based microarrays, we genotyped CNVs genome-wide in a birth cohort of 723 New Zealanders, and correlated them with four intelligence-related phenotypes. We found no significant association for any common CNV after false discovery correction, which is consistent with previous work. In contrast to a previous study, however, we found no effect on any cognitive measure of rare CNV burden, defined as total number of bases inserted or deleted in CNVs rarer than 5%. We discuss possible reasons for this failure to replicate, including interaction between CNV and aging in determining the effects of rare CNVs. While our results suggest that no CNV assayable by SNP chips contributes more than a very small amount to variation in human intelligence, it remains possible that common CNVs in segmental duplication arrays, which are not well covered by SNP chips, are important contributors.

## Introduction

A large body of work comparing monozygotic and dyzygotic twin sets indicates that approximately half of the variation in human intelligence as measured by standard tests is genetic, with estimates ranging between 40 and 80% [1], [2], [3], [4], [5], [6]. Intelligence is also remarkably tractable and stable, with different groups of cognitive tests ranking participants very similarly [7]. As is the case for many other heritable complex traits, however, individual genetic polymorphisms explaining substantial proportions of variation in intelligence-related phenotypes have not been identified despite multiple large-scale studies [8], [9], [10]. Perhaps surprisingly given the high heritability of testable intelligence, no robust or consistent link with any gene has yet been found in healthy individuals [11].

Prior to the emergence of high-throughput genotyping technologies, investigation of the genetics of intelligence focused on particular candidate genes selected on the basis of known aspects of gene function [12]. While many significant associations were found by this approach, these results were not replicated by studies from 2005 onwards that investigated thousands of polymorphisms across the whole human genome [13], [14], [15], [16], [17]. The most recent genome-wide association studies (GWAS), which have assayed several hundred thousand common single-nucleotide polymorphisms (SNPs) in as many as 11,000 people, have also not consistently identified any SNP significantly associated with intelligence after false discovery correction, and none which explains more than a tiny fraction of its inter-individual variation [8], [9], [10]. One plausible explanation for the failure of common SNPs to account for the heritability of intelligence is that a large number, perhaps many thousands, of genetic variants each contribute a very small amount [2], [8]. Similarly to most other complex traits, the extent to which this explanation is correct is still uncertain because other sources of genetic variation including rare SNPs, microsatellite length polymorphisms, gene-gene and gene-environment interactions, epigenetic modifications and copy number variations remain under-explored [18].

Since 2004 genome-wide microarray technology and whole genome sequencing have revealed that copy number variations (CNVs), deletions or duplications of sections of DNA as large as several mega bases, are present in all individuals and are responsible for a large proportion of normal human genetic variation, in fact more than SNPs in terms of numbers of variable nucleotides [19], [20], [21], [22]. Many CNV regions contain genes related to development and intelligence [23], [24], [25], and large, rare CNVs, have often been linked to schizophrenia, autistic spectrum disorder, and other developmental disorders with associated intellectual impairment [26], [27], [28], [29], [30], [31], [32]. Determining pathogenicity of particular CNVs in individuals affected by these disorders is often difficult or impossible because many variants increase risk while still being present in healthy controls [33], and in general the study of rare variants faces the problem that extremely large sample sizes are usually required to demonstrate statistically significant associations [18]. However, both autistic spectrum disorder and schizophrenia are significantly associated with elevated total amounts of rare CNV genome-wide (CNV burden) [30], [31], which can be correlated with phenotype without large sample sizes.

Body asymmetry, presumably reflecting mild developmental perturbation, is associated with reduced intelligence in individuals unaffected by clinically defined developmental disorders [34] and as many as 80% of mammalian genes are expressed in the brain [35]. These and other clues suggested the hypothesis that increased CNV burden may cause a reduction in cognitive ability in normal individuals, potentially via impaired development of neuronal structures [36].This was recently tested in 74 otherwise normal adults meeting criteria for alcohol dependence, and a significant correlation between IQ score, measured by the Wechsler abbreviated scale of intelligence [37] and CNV burden, defined as number of bases deleted in CNVs with frequencies of 5% or less, was found (p = 0.01) [36]. Here we report a failure to replicate this result in 717 individuals participating in the Christchurch Health and Development study (CHDS), a birth cohort consisting of children born in Christchurch New Zealand within a four-month period during mid-1977.

Common CNVs, i.e. variants with population frequencies greater than 5%, are also under-studied extensively in relation to cognitive ability. To our knowledge there is only one published GWAS, which examined 178 SNPs known to tag common CNVs in 1250 individuals, and correlated them with 11 intelligence-related phenotypes [38]. No significant associations were found after multiple hypothesis correction, but an enrichment of low P values encouraged further investigation. In addition to our study of rare CNV burden, we report a GWAS of common CNV regions versus scores on four different tests of intellectual ability and academic achievement in the CHDS cohort. We have also not identified any significant association after multiple hypothesis correction, providing further evidence that no individual CNV assayable by SNP-based chips has a substantial effect on intelligence in normal populations.

## Methods

### CNV Genotyping and Analysis

CNVs were detected using SNP-based Illumina 660W-Quad bead chips and called by PennCNV software following standard QC procedures [39]. Automatic QC of CNV calls was performed using program filter_cnv.pl from the PennCNV package with default settings: qcbafdrift (B allele frequency drift) wass set at 0.01 and qcwf (waviness factor) was set at 0.05. At least three consecutive positive probes were required for a CNV call.

Previously used standards of high individual CNV count, potentially indicating DNA degradation, are 40 [40] and 80 CNVs [41]. We used the lower threshold and excluded 137 individuals with more than 40 CNVs from our GWAS, leaving a sample size of 586. While the number of excluded individuals was clearly high, our DNA samples were not generally of poor quality, since they showed a failure rate of only 14 out of 761 in the SNP-based study performed on the same chip data, which was the lowest among the three cohorts used in the study. For the CNV burden tests, we repeated the analyses using the whole dataset, as well as the filtered set of 586 individuals, and this had no effect on the results.

Where CNVs overlapped, we used standard modules of the Plink software package (version 1.07) to define them as identical or separate, and to assign each CNV a frequency within the dataset. Following Yeo et al., (2011) [36], we used 5% frequency as the dividing line between rare and common CNVs. For our GWAS we merged overlapping common CNVs into regions and scored these regions as CNV or not CNV in each individual.

### Tests of Intellectual Ability

Our test subjects were 723 individuals participating in the Christchurch Health and Development study (CHDS), a birth cohort consisting of children born in the Christchurch New Zealand urban area within a four-month period during mid-1977. The participants have been assessed at regular intervals using various biological and behavioural measures [42], [43]. Here we have correlated CNVs with the following four tests of cognitive ability/academic achievement:

#### WISC-R total IQ (8–9 years)

Child cognitive ability was assessed at ages 8, 9 years using the revised Wechsler Intelligence Scale for Children [44]. Assessments were based on four verbal and four performance subscales administered at each age. The measure used in the present analysis is the average of the child’s estimated total IQ scores from each assessment. (Mean IQ = 104.7, SD = 13.9, range 63–144).

#### The test of scholastic abilities (13 years)

The Test of Scholastic Abilities (TOSCA) provides a measure of the extent to which a child exhibits the skills and competencies necessary to deal with the academic requirements of high school education [45] (Mean TOSCA score = 36.4, SD = 14.8, range 0–69).

#### Burt word reading test (18 years)

The Burt Word Reading Test [46] provides a generalised measure of word recognition ability. The test was administered at age 18, and the total score calculated based on the number of words correctly identified. (Mean Burt = 98.2, SD = 12.2, range 26–110).

#### Overall academic achievement (25 years)

On the basis of participant reports of their educational history and attainment of qualifications sample members were classified on a 7-point scale reflecting their highest level of academic attainment by age 25 years [47]. This scale ranged from 1 = no formal qualifications to 7 = attained a university degree. (Mean achievement score = 4.4, SD, SD = 2.2).

Assessment of cognitive outcomes up to age 13 was limited to the sub-sample of the cohort resident in the Canterbury region (approximately 80% of the total available cohort at each age). As a result the sample sizes assessed on the WISC-R and TOSCA are somewhat lower than for the measures assessed in young adulthood.

### Alcohol Dependence

At ages 18, 21, 25 and 30 participants were questioned about patterns of alcohol use/misuse since the previous assessment. As part of this questioning relevant items from the Composite International Diagnostic Interview [48] were used to assess DSM-IV [49] diagnostic criteria for alcohol dependence. Participants were classified as having alcohol dependence if they met diagnostic criteria for dependence at any time from age 18–30 years.

### Ethical Approval

All phases of the CHDS were subject to ethical approval from the Canterbury (NZ) Regional Health and Disability Ethics Committee and all aspects of data collection were with the written consent of research participants.

## Results

In 723 individuals we found a total of 22,249 CNVs with a mean length of 60,867 base pairs (bp). Locations and genotypes of all CNVs called can be found in Table S1. We made a division between rare and common CNVs with a threshold of 5% frequency. With the rare CNVs, we performed an analysis of total CNV burden, in terms of number of bases deleted or inserted per individual, versus scores on four different tests of intellectual ability. We also performed a GWAS of common CNVs, first combining overlapping CNVs into CNV regions (CNVRs). There were 53 CNVRs with greater than 5% frequency in our sample, and we evaluated the effect of each of these against our cognitive test scores using Pearson parametric correlation.

### Rare CNV Burden Analysis

We first attempted to replicate the results of Yeo et al., (2011) [36] who found a significant negative correlation between IQ score and total number of bases deleted in CNVs below 5% frequency [36]. In our filtered sample, individuals lost an average of 309,618 bp (SD = 316,857) in 12.15 rare deletions (SD = 6.5). These numbers are somewhat higher than those in the sample studied by Yeo and colleagues [36], which likely reflects platform-specific differences in sensitivity of CNV detection (Ronald Yeo, personal communication). Table 1 shows the correlations between selected measures of cognitive ability/academic achievement and the number/size of rare CNVs in the CHDS cohort. The measures of ability/achievement were selected to span the age range from childhood to adulthood and included: 1) the full scale WISC-R total IQ score assessed at ages 8–9 years; 2) the Test of Scholastic Abilities (TOSCA) administered at age 13 years; 3) the Burt Word Reading Test administered at age 18 years; and 4) a global measure of academic achievement by age 25 years. We selected these measures from a much broader array of cognitive/academic outcomes, all of which exhibit the same general properties in terms of absence of association with CNVs.

**Table 1:** Showing Pearson correlations between measures of cognitive ability and the number and size of rare CNVs.

Measure	WISC-R Total IQScore (8–9 years)	Test of ScholasticAbilities (TOSCA) (13 years)	Burt Word ReadingTest (18 years)	Overall AcademicAchievement (25 years)
**Total sample**				
Length of rare deletions	.02	.04	−.00	.02
Length of rare insertions	−.02	−.03	.02	−.03
Number of rare CNVs	.03	.05	.01	.02
	(N = 567)	(N = 527)	(N = 697)	(N = 717)
**Filtered sample excluding those with high (40+) CNV count**				
Length of rare deletions	−.04	.03	−.00	.03
Length of rare insertions	−.02	−.01	.04	−.01
Number of rare CNVs	.02	.04	.04	.06
	(N = 462)	(N = 431)	(N = 566)	(N = 581)
**Filtered sample meeting criteria for alcohol dependence**				
Length of rare deletions	.09	.10	.04	.01
Length of rare insertions	−.07	−.06	.01	.04
Number of rare CNVs	.03	−.02	.04	.06
	(N = 64)	(N = 56)	(N = 83)	(N = 87)

* All correlations statistically non-significant (p>.10).

For each outcome the table shows the observed Pearson correlations with three measures of rare CNV status: i) the length of rare deletions; ii) the length of rare insertions; and iii) the total number of rare CNVs. The correlations are estimated for each of three samples: i) the total available gene chip sample assessed on each cognitive/academic outcome; ii) the sample filtered to exclude subjects with a high CNV count (over 40 CNVs); and iii) the subset of the filtered sample who met DSM-IV diagnostic criteria for alcohol dependence from age 18–30 years. None of these correlations was statistically significant (P>0.1).

To examine the extent to which the findings may have been influenced by the inclusion of subjects with moderate or severe cognitive impairment, we re-analysed the data excluding all subjects who scored more than two standard deviations below the mean on the cognitive test measures. The resulting correlations were negligibly different from those reported in Table 1. Further analysis of residuals to identify and exclude a small number of potential outliers also had little impact on the observed pattern of correlations. In two cases the exclusion of outliers resulted in the observed correlation increasing to the point of statistical significance (p<0.05): these cases involved the correlation of academic achievement with length of rare deletions (r = .11) and number of rare CNVs (r = .10) in the sample filtered to exclude those with high CNV count. These exceptions aside, the correlations including or excluding outliers were otherwise very similar.

### GWAS on Common CNV Regions

Table 2 summarizes our findings from examination of correlations between cognitive/academic outcomes and the 53 CNVRs with more than 5% frequency in the dataset. The analysis suggests an almost complete absence of association between the CNV measures and each outcome. In each case tests of the distribution of the correlations against a normal distribution using Q-Q plots and goodness of fit tests showed that the distributions were consistent with random sampling from a normal distribution with mean zero.

**Table 2:** Summarizing the distribution of correlations between measures of cognitive ability/academic achievement and 53 CNVRs.

Cognitive/academic outcome	Mean (SD) Correlation	Range of Correlations	CNVRs significantly associated with outcome	
			Without Bonferroni correction (p<.05)	With Bonferroni correction (p<.0009)
WISC-R Total IQ score (8–9 years,N = 462)	.005 (.044)	−.088, .113	CNVR50 (r = .11)	
TOSCA (13 years, N = 431)	−.000 (.051)	−.110, .114	CNVR5 (r = −.11); CNVR17 (r = −.10); CNVR43 (r = −.11); CNVR49 (r = .10); CNVR50 (r = .11)	
Burt Word Reading Test (18 years, N = 566)	−.002 (.041)	−.081, .063		
Overall academic achievement(25 years, N = 581)	.007 (.050)	−.120, .144	CNVR17 (r = −.12); CNVR46 (r = .14); CNVR50 (r = .09)	CNVR46 (r = .14)

Overall nine correlations were statistically significant at the p<0.05 level, no more than would be expected by chance. Five of these correlations were accounted for by two CNVRs: CNVR50 showed significant positive correlations with three of the four outcomes, and CNVR17 significant negative associations with two outcomes. However, after application of a Bonferroni correction for multiple comparisons only one correlation (CNVR46 and academic achievement, r = .14, p = 0.0005) reached significance using the Bonferroni corrected p value (p = 0.0009), in association with overall academic achievement at age 25. Further examination showed that CNVR46 had negative and non-significant correlations with other cognitive/achievement outcomes, suggesting that this correlation could easily reflect chance variation in the data. None of the nominally significant CNVRs overlapped with any SNP listed as being significant at any level by three previous large GWAS on cognitive ability [8], [9], [10]. Locations of the nominally significant CNVRs and their gene contents can be found in Table S2. Pseudogenes and genes of unknown function are the most common gene types involved, and the remainder, including low density lipoprotein and olfactory receptors, are of no obvious relevance to cognitive ability.

Reanalysis of the data to exclude potential outliers or those with moderate/severe cognitive impairment did not substantively alter these findings. In each case the observed pattern of correlations was consistent with those reported in Table 2. While a small number of correlations were nominally significant at p<.05, no correlation other than that between CNVR46 and academic achievement exceeded the Bonferroni corrected significance value (p<.0009).

## Discussion

Our findings do not provide strong evidence for an association between any common CNV region and cognitive/academic performance scores, and we were unable to detect any effect of rare CNV burden on these outcomes. Given previous evidence that CNVs can affect intelligence-related phenotypes, the most obvious explanation for our negative results is that we didn’t sample a sufficient number of functional CNVs. In view of the fact that we couldn’t detect any enrichment of non-significant correlations, we cannot argue that testing a larger number of individuals by our methods would reveal significant associations. However, it is likely that our chip probes missed some important variants, particularly segmental duplication arrays, as these are not easily assayable by SNP chips or other standard genotyping methods [50], [51], [52]. One example of this is the CNV encoding repeats of a protein domain of unknown function called DUF1220 on Chromosome 1q21.1 [53]. The size of this duplication array correlates well with brain size between species [23], [54], as well as within the human population [55], [56], but because it has increased dramatically in copy number during the evolution of the primate lineage it has too many copies to assay accurately by normal high-throughput methods. Consistent with the previous published GWAS of CNV and cognitive measures, which also didn’t identify any significant associations after false discovery correction [38], our results encourage focus on these less accessible CNVs.

In general, common variants are expected to have smaller phenotypic effects than rare ones [57] and CNVs that have been associated with intellectual and developmental impairment are predominantly large, rare *de novo* mutations rather than commonly segregating alleles [28], [29], [58]. It’s therefore less clear why we were unable to replicate the significant association found by Yeo and colleagues between rare CNV burden and intelligence [36]. Our much larger sample size suggests that our results should be considered more reliable, but methodological differences may be relevant. A recent large twin study reported that heritability of general cognitive ability increases significantly and linearly from 41% in childhood (9 years) to 55% in adolescence (12 years) and to 66% in young adulthood (17 years) [3], and strong evidence is emerging that genetics makes a substantial contribution to changes in intelligence from childhood to old age [59]. Yeo et al., (2011) [36] tested IQ score at ages ranging between 22 and 55, and our measures were taken at 8–9, 13, 18 and 25 years, with IQ only sampled at ages 8–9. It may therefore be that the rare CNVs that caused the association they found were related to an interaction between CNV, age and cognitive ability, and future studies should account for this possibility by including age as a co-factor or stratifying samples by age group. Although their sample was restricted to alcohol-dependent individuals, a substantial contribution by alcoholism is unlikely since we found no significant correlations for the subset of our cohort meeting diagnostic criteria for alcohol dependence (DSM-IV) [49], albeit with small sample sizes of 64–87 individuals.

Theoretically, it is also possible that population-specific variants were involved. However, while our study participants were born in New Zealand and Yeo et al. (2011) [36] studied North Americans, the subset of their sample showing the largest association with rare CNV burden was the 45% classified as Anglo/White, and these individuals should be more similar to our cohort, the majority of whom are of British descent, than to other ethnicities in their sample. Effects of sex also do not explain the discrepancy, since although Yeo and colleagues [36] sampled 53 males and only 21 females, we found no difference between the sexes in the effect of CNV burden (data not shown).

In conclusion, our results are in line with previous work showing that individual common variants assayable by SNP chips do not have strong effects on cognitive ability, and suggest that total burden of rare CNVs is also not a major contributor. There are currently more promising approaches to elucidating the genetic component of intelligence. These include larger studies to identify individual functional rare variants, new statistical methods allowing evaluation of the effects of agglomerations of common variants [8], and testing of other under-studied sources of genetic variation such as promoter microsatellites and segmental duplication arrays, as well as gene-gene and gene-environment interactions.

## Supporting Information

Table S1 **All CNV data. Genomic coordinates are HG18.**
(XLS)

Table S2 **Locations and gene contents of CNVRs associated with cognitive measures at a nominal level (P<0.05).** No P values were significant after false discovery correction. Coordinates are HG18.
(XLS)
